# Differences in TAVR Utilization in Aortic Stenosis Among Patients With and Without Psychiatric Comorbidities

**DOI:** 10.1016/j.jscai.2024.102235

**Published:** 2024-08-13

**Authors:** Mohamed Abugrin, Alsu Zagorulko, Batoul Aboulqassim, Ahmad Raja, Harshith Thyagaturu, Ahmed Khadra, Vikrant Jagadeesan, Pavel Sinyagovsky

**Affiliations:** a Department of Internal Medicine, Bassett Medical Center, Cooperstown, New York; b Department of Medicine, Russian National Research Medical University, Moscow, Russia; c Faculty of Medicine, University of Tripoli, Tripoli, Libya; d Department of Cardiology, West Virginia University Heart and Vascular Institute, Morgantown, West Virginia; e Department of Internal Medicine, Yuma Regional Medical Center, Yuma, Arizona

**Keywords:** aortic stenosis, depression, disparity, mental health, transcatheter aortic valve replacement

## Abstract

**Background:**

Transcatheter aortic valve replacement (TAVR) is one of the primary treatment modalities for aortic stenosis (AS). Disparities affecting certain groups could result in lower utilization of this life-saving procedure. This study aims to investigate the effects of associated psychiatric conditions on the likelihood of TAVR in hospitalized AS patients.

**Methods:**

Our retrospective observational study used the National Inpatient Sample to identify hospitalized patients with AS. Using the International Classification of Diseases, 10th Revision, Clinical Modification patients were stratified into those without psychiatric comorbidities, and those with psychiatric comorbidities. The primary outcome was comparing the odds of TAVR between AS patients with and without psychiatric comorbidities. The secondary outcome assessed the association between TAVR and specific psychiatric comorbidities, using multivariable logistic regression while adjusting for prespecified covariates.

**Results:**

The study included 1,549,785 AS patients, of which 26% had psychiatric comorbidities. Patients with any psychiatric comorbidity had a significantly reduced likelihood of TAVR (adjusted odds ratio [aOR], 0.76; *P* < .001). For 2 psychiatric comorbidities, (aOR, 0.80; *P* < .001), and for more than 2 comorbid mental disorders (aOR, 0.46; *P* < .001). Lower TAVR odds were observed in patients with depression (aOR, 0.79), anxiety (aOR, 0.79), bipolar disorder (aOR, 0.74), substance use (aOR, 0.73), and psychotic disorders (aOR, 0.61), with *P* values < .001. There was no significant difference in the odds of surgical aortic valve replacement between those with and without psychiatric comorbidities.

**Conclusions:**

AS patients with psychiatric conditions face reduced TAVR likelihood. Further research is needed to confirm, explore, and address factors contributing to this disparity.

## Introduction

Aortic stenosis (AS) is a frequently encountered valvular heart disease that poses a significant morbidity and mortality risk when it reaches a severe stage.^1^, ^2^, ^3^ The primary treatment modalities for severe AS are transcatheter aortic valve replacement (TAVR) and surgical aortic valve replacement (SAVR), each offering its own set of advantages and considerations. The decision between TAVR and SAVR is highly individualized, taking into account the patient’s age, life expectancy, valve anatomy, frailty, cardiac condition, and surgical risk.^4^

TAVR has shown great efficacy in improving patient outcomes, with a focus on reducing symptoms, enhancing overall quality of life, and decreasing mortality rates.^5^, ^6^, ^7^, ^8^, ^9^ However, despite these advancements, there exists concerning underutilization of TAVR, particularly among certain racial/ethnic groups, which results in lower utilization of this life-saving procedure.^10^, ^11^, ^12^

Psychiatric conditions have a significant prevalence in the United States, affecting approximately 18% of adults annually.^13^ These patients experience higher all-cause mortality rates compared to the general population and encounter numerous disparities in health care delivery.^14^ Among these disparities is a reduced likelihood of receiving guideline-directed medical therapy, as well as lower rates of medical procedures such as percutaneous coronary intervention, coronary artery bypass grafting, carotid endarterectomy, cerebrovascular arteriography, tissue plasminogen activator administration, and cerebral revascularization.^15^, ^16^, ^17^, ^18^, ^19^, ^20^, ^21^

This study aims to shed more light on potential disparities in treatment. Specifically, it investigates whether the presence of psychiatric conditions similarly affects the likelihood of undergoing TAVR in patients with AS.

## Methods

### Study design and data source

We conducted an observational retrospective cohort study utilizing data from the National Inpatient Sample (NIS), a component of the Healthcare Cost and Utilization Project (HCUP). The NIS is the largest all-payer inpatient care database in the United States, capturing approximately a 20% sample of US hospitals. It encompasses data from about 1000 hospitals, accounting for over 8 million hospital stays annually. The unit of observation is the inpatient stay record, which contains patient demographic details (age, sex, race, median household income for ZIP codes, comorbidities, etc) and hospital-level characteristics (hospital bed size, location, teaching status, discharge status, etc). Discharge-level data are weighted and used to calculate national estimates. Comorbidities are coded using the International Classification of Diseases, 10th Revision, Clinical Modification (ICD-10-CM). These data are deidentified and publicly available at the Agency for Healthcare Research and Quality (www.hcup-us.ahrq.gov). Therefore, institutional review board approval was not required for our study.

### Identification of study cohort

**Figure 1:**
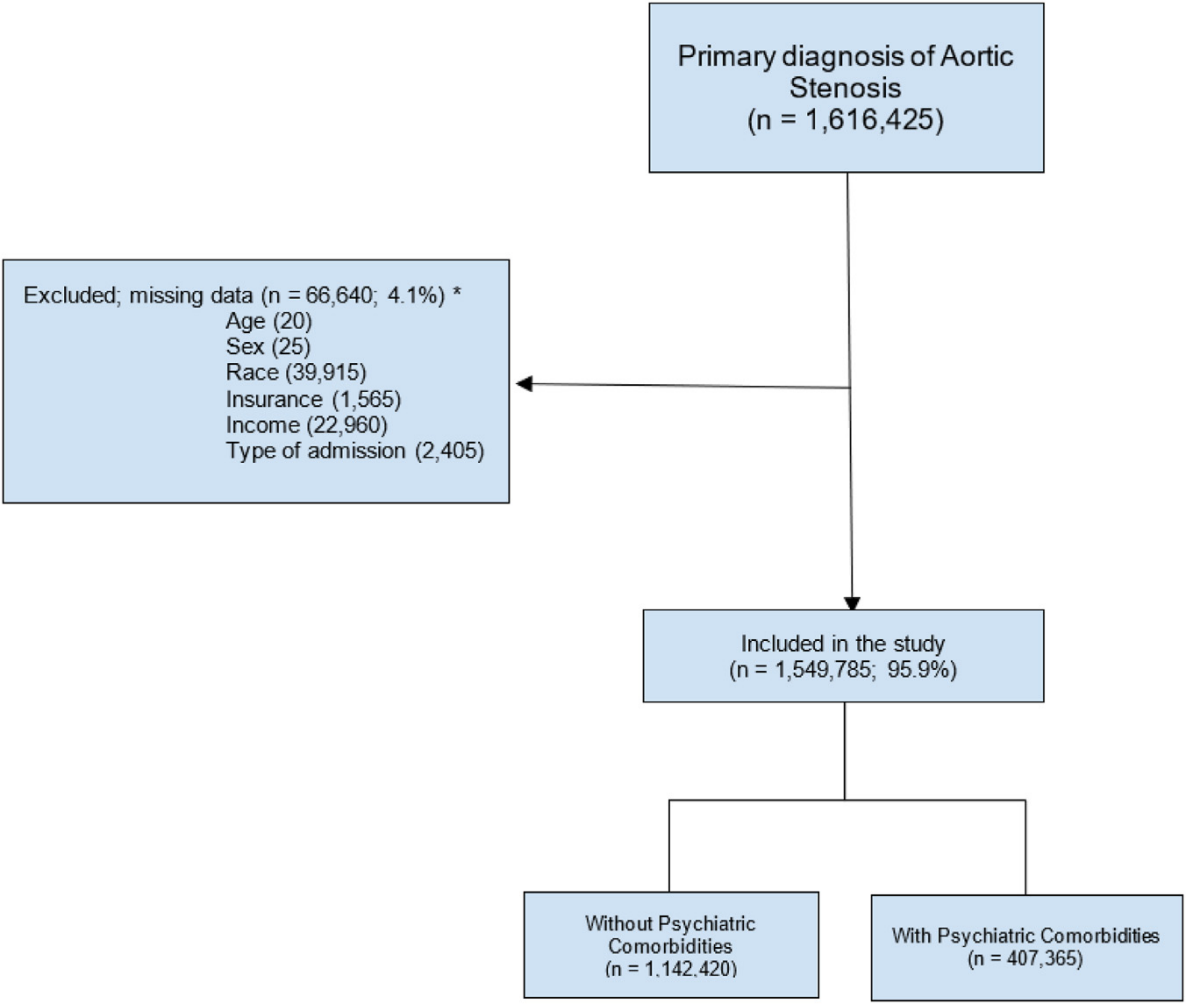
**Flow diagram for case selection.** ∗Categories not mutually exclusive. n, number.

We identified hospitalized adults (>18 years) with a primary diagnosis of AS from January 2017 to December 2019 using ICD-10-CM diagnosis codes (I350, I352, I358). The exposure of interest was the presence of comorbid psychiatric conditions such as depression, bipolar disorder, anxiety, substance use disorder, schizophrenia, or other psychoses as described previously.^20^, ^21^, ^22^, ^23^ These comorbidities were identified using relevant ICD-10 codes, as outlined in the Clinical Classification Software (a complete list of ICD-10-CM codes used in this study is detailed in Supplemental Table S1). Multiple codes within the same diagnostic category were considered as a single psychiatric comorbidity. The psychiatric conditions could have been diagnosed in both inpatient and outpatient settings but were required to be captured in the inpatient stay record at discharge. Patient encounters were categorized into 2 groups: group 1 included those with ICD-10-CM codes for AS only, and group 2 comprised those with ICD-10-CM codes for both AS and psychiatric disorder. Records with missing information such as age, sex, race, income, insurance, and hospital characteristics were excluded. However, the total proportion of missing values was <5% (Figure 1).

### Primary and secondary outcomes

The primary outcome was the adjusted odds ratio (aOR) of undergoing TAVR, identified by ICD-10-PCS codes (02RF38H, 02RF38Z, 02RF3KH, 02RF3KZ), in patients with AS who have psychiatric comorbidities compared to those without psychiatric comorbidities. The secondary outcomes included assessing the association between TAVR and specific psychiatric disorders, as well as evaluating the cumulative impact of multiple psychiatric comorbidities on the likelihood of undergoing TAVR. Additionally, we examined the relationship between SAVR and psychiatric disorders.

### Statistical analysis

We conducted a comparative analysis between individuals with and without psychiatric conditions, evaluating demographic, clinical, and hospital characteristics. In accordance with HCUP regulations (https://hcup-us.ahrq.gov/db/nation/nis/nisdbdocumentation.jsp), analysis was conducted through appropriate stratifying, clustering, and weighting of samples (https://hcup-us.ahrq.gov/db/nation/nis/nischecklist.jsp). NIS-provided discharge weights were applied to all analyses to generate national estimates for this study. Categorical variables were analyzed using χ^2^ tests, and continuous variables were analyzed using Wilcoxon rank-sum tests adjusted for complex survey design. We utilized multivariable logistic regression models to evaluate the association between TAVR and psychiatric comorbidities, adjusting for covariates such as race, age, sex, region, health care system type, insurance type, admission type, household income, and clinical characteristics (a complete list of covariates included in multivariate analysis is detailed in Supplemental Table S2). The Charlson comorbidity index (CCI) was utilized to adjust for comorbidities. The CCI considers various health conditions such as heart disease, diabetes, kidney disease, liver disease, cancer, HIV/AIDS, and more. This index offers a standardized method to assess the influence of comorbidities on prognosis. To mitigate potential bias from unmeasured and excluded variables, subpopulation and sensitivity analyses on covariates such as race and sex were conducted. Additionally, we examined interactions between psychiatric comorbidity and sex, as well as psychiatric comorbidity and race, in relation to TAVR. Statistical significance was determined at *P* < .05, with 95% CI reported. All statistical analyses in this study were performed using R software (version 4.2.3, R Foundation for Statistical Computing). For a more detailed description of the statistical models used in our analysis, refer to the supplementary material.

## Results

### Sample characteristics

The primary cohort consisted of 1,549,785 patient encounters with AS, of which 407,365 (26%) had any of the 5 psychiatric disorders of interest. The median age of patients was 80 years (IQR, 72-87; *P* < .001). TAVR was performed in 25,660 (6.3%) of encounters involving individuals with psychiatric disorders, compared to 116,440 (10%) of those without psychiatric disorders. SAVR was performed in 25,035 (6.1%) of encounters with psychiatric disorders, which is comparable to the 69,345 (6.1%) in those without psychiatric disorders. Additional demographic, clinical, and hospital characteristics of the study cohort, stratified by the presence of psychiatric conditions, are presented in Table 1.

**Table 1:** Baseline characteristics of the study population stratified by the presence of any comorbid psychiatric comorbidities.

Characteristics	No psychiatric comorbidities (n = 1,142,420)	Psychiatric comorbidities (n = 407,365)	*P* value
Age, y	81 (73, 87)	76 (67, 84)	<.001
Female	537,990 (47%)	218,005 (54%)	<.001
Race			<.001
White	934,795 (82%)	345,980 (85%)	–
Asian or Pacific Islander	23,455 (2.1%)	3,820 (0.9%)	–
Black	80,130 (7.0%)	26,830 (6.6%)	–
Hispanic	75,020 (6.6%)	22,385 (5.5%)	–
Native American	3615 (0.3%)	1475 (0.4%)	–
Other	25,405 (2.2%)	6875 (1.7%)	–
Insurance			<.001
Medicare	989,070 (87%)	329,295 (81%)	–
Medicaid	25,680 (2.2%)	21,125 (5.2%)	–
No charge	720 (<0.1%)	540 (0.1%)	–
Other	16,225 (1.4%)	7,245 (1.8%)	–
Private insurance	102,835 (9.0%)	43,650 (11%)	–
Self-pay	7890 (0.7%)	5510 (1.4%)	–
Type of admission			<.001
Elective admission	275,215 (24%)	79,750 (20%)	–
Nonelective admission	867,205 (76%)	327,615 (80%)	–
Hospital region			<.001
Midwest	265,680 (23%)	107,825 (26%)	–
Northeast	271,645 (24%)	87,555 (21%)	–
South	393,250 (34%)	141,865 (35%)	–
West	211,845 (19%)	70,120 (17%)	–
Hospital control			.3
Government nonfederal	97,630 (8.5%)	35,625 (8.7%)	–
Private invest-own	137,370 (12%)	48,235 (12%)	–
Private not-profit	907,420 (79%)	323,505 (79%)	–
Hospital teaching status and location			.002
Rural	83,740 (7.3%)	31,920 (7.8%)	–
Urban nonteaching	226,365 (20%)	80,120 (20%)	–
Urban teaching	832,315 (73%)	295,325 (72%)	–
Hospital bed size			.11
Large	599,130 (52%)	211,165 (52%)	–
Medium	329,915 (29%)	118,840 (29%)	–
Small	213,375 (19%)	77,360 (19%)	–
Household income quartile			<.001
0 to 25th percentile	263,290 (23%)	105,300 (26%)	–
26th to 50th percentile (median)	296,755 (26%)	108,640 (27%)	–
51st to 75th percentile	297,520 (26%)	104,820 (26%)	–
76th to 100th percentile	284,855 (25%)	88,605 (22%)	–
Charleston comorbidity index	3.00 (2.00, 5.00)	4.00 (3.00, 5.00)	<.001
Dementia	160,480 (14%)	64,045 (16%)	<.001
Chronic kidney disease	455,170 (40%)	140,775 (35%)	<.001
Diabetes mellitus	456,690 (40%)	159,385 (39%)	<.001
Essential hypertension	307,450 (27%)	118,715 (29%)	<.001
Hyperlipidemia	708,710 (62%)	256,705 (63%)	<.001
Atrial fibrillation	502,115 (44%)	151,905 (37%)	<.001
Peripheral artery disease	126,145 (11%)	52,175 (13%)	<.001
Bicuspid aortic valve	7220 (0.6%)	3305 (0.8%)	<.001

Values are n (%) or median (IQR) unless otherwise indicated. All baseline characteristics were included in the covariate analysis.

### Primary outcome

Multivariable analysis revealed that AS patients with psychiatric comorbidities have lower odds of undergoing TAVR when compared to AS patients without psychiatric comorbidities (aOR, 0.76; 95% CI, 0.72-0.81; *P* < .001). There were no statistically significant interactions between race or sex and psychiatric comorbidities in the odds of undergoing TAVR (*P* > .05).

### Secondary outcome

#### Comparison of TAVR utilization based on psychiatric comorbidities

In comparison to patient encounters without any psychiatric comorbidities, the presence of 1 psychiatric comorbidity was associated with a lower likelihood of receiving TAVR (aOR, 0.76; 95% CI, 0.72-0.81; *P* < .001). Similarly, the presence of 2 psychiatric comorbidities had lower odds of TAVR utilization (aOR, 0.80; 95% CI, 0.73-0.89;*P* < .001). The presence of more than 2 comorbid mental disorders was associated with significantly lower odds of receiving TAVR (aOR, 0.46; 95% CI, 0.33-0.64; *P* < .001)

#### Comparison of TAVR utilization based on specific psychiatric disorder

**Figure 2:**
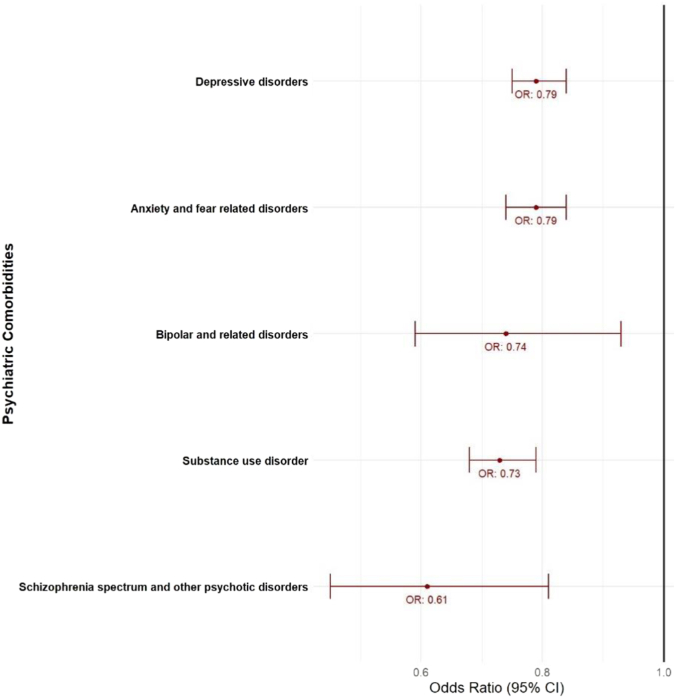
**Adjusted odds ratios of transcatheter aortic valve replacement in aortic stenosis patients with various psychiatric disorders.** OR, odds ratio.

When comparing patient encounters with specific psychiatric comorbidities to those without any mental disorders, lower odds of TAVR utilization were observed in the following groups: depressive disorders (aOR, 0.79; 95% CI, 0.75-0.84; *P* < .001), anxiety and fear-related disorders (aOR, 0.79; 95% CI, 0.74-0.84; *P* < .001), bipolar and related disorders (aOR, 0.74; 95% CI, 0.59-0.93; *P* = .011), substance use disorder (aOR, 0.73; 95% CI, 0.68-0.79; *P* < .001), and schizophrenia spectrum and other psychotic disorders (aOR, 0.61; 95% CI, 0.45-0.81; *P* < .001). The forest plot is presented in Figure 2.

**Central Illustration:**
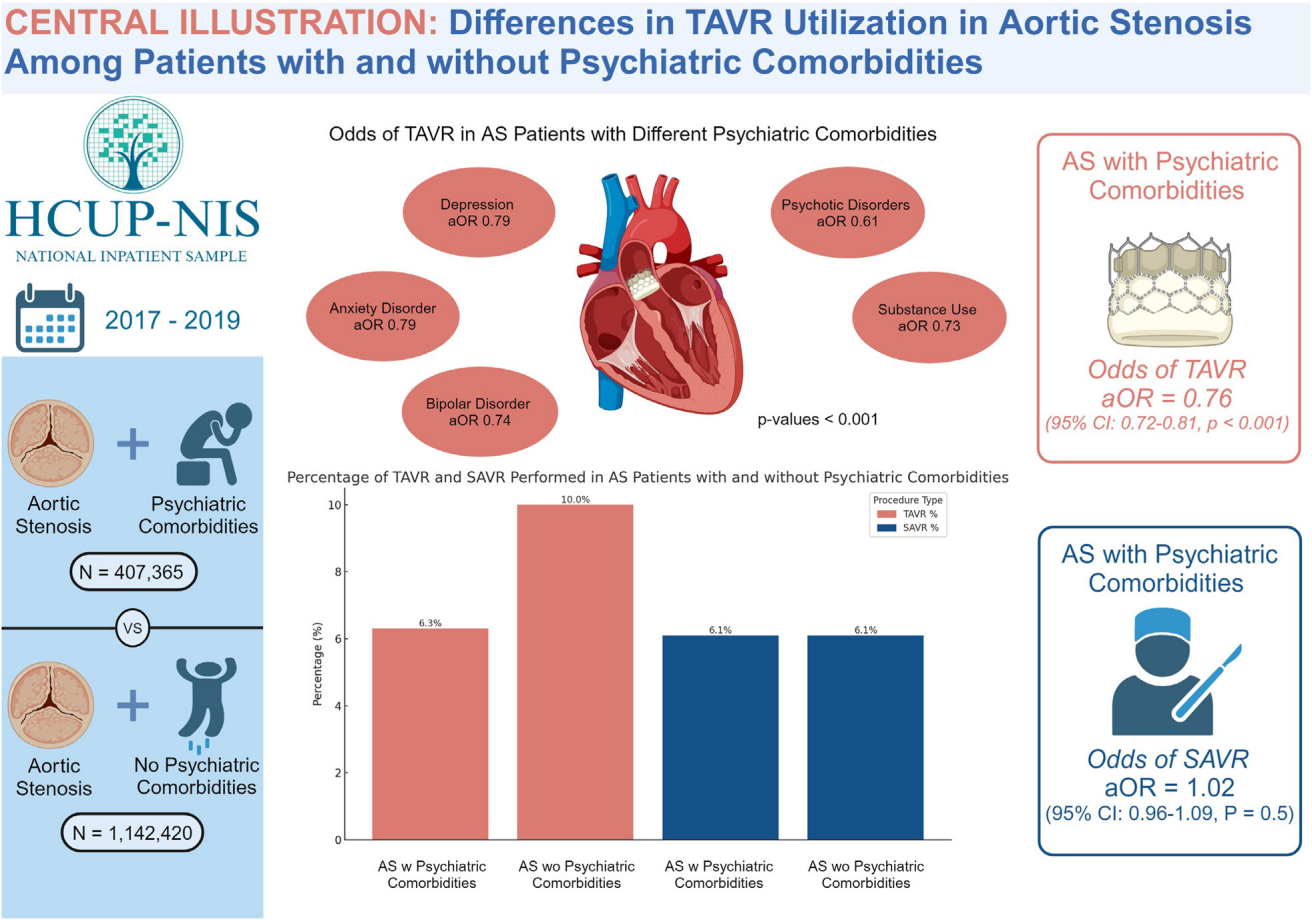
**Differences in transcatheter aortic valve replacement (TAVR) utilization in aortic stenosis (AS) among patients with and without psychiatric comorbidities.** aOR, adjusted odds ratio; HCUP-NIS, Healthcare Cost and Utilization Project National Inpatient Sample; N, number; SAVR, surgical aortic valve replacement; w, with; wo, without.

#### Covariates associated with TAVR

In adjusted models, significantly lower odds of TAVR were observed in females (aOR, 0.86; 95% CI, 0.81-0.92; *P* < .001), Blacks (aOR, 0.62; 95% CI, 0.53-0.72; *P* < .001), and Asians or Pacific Islanders (aOR, 0.51; 95% CI, 0.39-0.68; *P* < .001). Additional covariates included in the analysis are presented in Table 2.

**Table 2:** Adjusted odds of covariates associated with TAVR.

Characteristic	Adjusted odds ratio	95% CI	*P* value
Age	1.00	1.00-1.01	.013
Sex			
Male	Reference		
Female	0.86	0.81-0.92	<.001
Race			
White	Reference		
Asian or Pacific Islander	0.51	0.39-0.68	<.001
Black	0.62	0.53-0.72	<.001
Hispanic	0.86	0.67-1.12	.3
Native American	1.30	0.80-2.11	.3
Other	1.33	1.02-1.73	.037
Insurance			
Medicare	Reference		
Medicaid	0.72	0.57-0.91	.006
No charge	0.94	0.37-2.40	.9
Other	1.29	1.00-1.66	.050
Private insurance	0.75	0.64-0.89	.001
Self-pay	0.67	0.45-0.99	.044
Hospital region			
Midwest	Reference		
Northeast	1.85	1.50-2.29	<.001
South	1.73	1.39-2.16	<.001
West	1.68	1.32, 2.13	<.001
Hospital control			
Government nonfederal	Reference		
Private invest-own	1.84	1.26-2.68	.001
Private not-profit	0.97	0.69-1.36	.9
Hospital teaching status and location			
Rural	Reference		
Urban nonteaching	4.15	2.20-7.84	<.001
Urban teaching	24.9	14.0-44.3	<.001
Hospital bed size			
Small	Reference		
Large	5.57	4.12-7.52	<.001
Medium	1.64	1.17-2.31	.004
Household income quartile			
0 to 25th percentile	Reference		
26th to 50th percentile (median)	1.06	0.95-1.18	.3
51st to 75th percentile	1.04	0.92-1.17	.5
76th to 100th percentile	1.07	0.94-1.22	.3
Charleston comorbidity index	0.99	0.96-1.02	.5
Comorbidities			
Dementia	0.43	0.39-0.48	<.001
Chronic kidney disease	0.88	0.81-0.97	.008
Diabetes mellitus	0.96	0.89-1.03	.2
Essential hypertension	0.39	0.34-0.45	<.001
Hyperlipidemia	1.47	1.36-1.60	<.001
Atrial fibrillation	0.87	0.81-0.93	<.001
Peripheral artery disease	1.47	1.33-1.63	<.001
Bicuspid aortic valve	1.39	0.90-2.15	.14

aOR, adjusted odd ratio; TAVR, transcatheter aortic valve replacement.

#### SAVR utilization and psychiatric disorders

Multivariable analysis revealed no significant difference in SAVR utilization between AS patients with and without psychiatric comorbidities (aOR, 1.02; 95% CI, 0.96-1.09; *P* = .5). When comparing patients with different numbers of psychiatric comorbidities to those without, the following associations were observed: 2 comorbidities (aOR, 0.97; 95% CI, 0.87-1.09; *P* = .7), and more than 2 comorbidities (aOR, 1.14; 95% CI, 0.90-1.43; *P* = .3). There was no significant association between SAVR and the presence of 2 or more psychiatric comorbidities in those with AS. Table 3 presents the demographic, clinical, and hospital characteristics of patients who underwent SAVR compared to TAVR.

**Table 3:** Demographic, clinical, and hospital characteristics of patients who underwent SAVR compared to TAVR.

Characteristic	SAVR n = 94,245	TAVR n = 142,100	*P* value
Age, y	70 (62, 75)	81 (74, 86)	<.001
Female	29,760 (32%)	63,955 (45%)	<.001
Race			<.001
White	80,050 (85%)	124,575 (88%)	–
Asian or Pacific Islander	1260 (1.3%)	1770 (1.2%)	–
Black	3580 (3.8%)	5660 (4.0%)	–
Hispanic	6715 (7.1%)	6675 (4.7%)	–
Native American	365 (0.4%)	415 (0.3%)	–
Other	2275 (2.4%)	3005 (2.1%)	–
Insurance			<.001
Medicare	61,220 (65%)	127,330 (90%)	–
Medicaid	4380 (4.6%)	1730 (1.2%)	–
No charge	125 (0.1%)	45 (<0.1%)	–
Other	2040 (2.2%)	2425 (1.7%)	–
Private insurance	25,045 (27%)	10,025 (7.1%)	–
Self-pay	1435 (1.5%)	545 (0.4%)	–
Type of admission			<.001
Elective admission	72,785 (77%)	120,105 (85%)	–
Nonelective admission	21,460 (23%)	21,995 (15%)	–
Hospital region			.016
Midwest	22,555 (24%)	32,285 (23%)	–
Northeast	19,235 (20%)	33,490 (24%)	–
South	34,155 (36%)	48,305 (34%)	–
West	18,300 (19%)	28,020 (20%)	–
Hospital control			<.001
Government nonfederal	6735 (7.1%)	10,975 (7.7%)	–
Private invest-own	11,375 (12%)	13,575 (9.6%)	–
Private not-profit	76,135 (81%)	117,550 (83%)	–
Hospital teaching status and location			<.001
Rural	2020 (2.1%)	1505 (1.1%)	–
Urban nonteaching	12,140 (13%)	12,410 (8.7%)	–
Urban teaching	80,085 (85%)	128,185 (90%)	–
Hospital bed size			<.001
Small	9695 (10%)	9455 (6.7%)	–
Large	61,710 (65%)	103,760 (73%)	–
Medium	22,840 (24%)	28,885 (20%)	–
Household income quartile			<.001
0 to 25th percentile	21,525 (23%)	29,675 (21%)	–
26th to 50th percentile (median)	25,735 (27%)	36,080 (25%)	–
51st to 75th percentile	25,325 (27%)	38,250 (27%)	–
76th to 100th percentile	21,660 (23%)	38,095 (27%)	–
Bicuspid aortic valve	6615 (7.0%)	715 (0.5%)	<.001
Depressive disorders	8470 (9.0%)	11,680 (8.2%)	<.001
Schizophrenia spectrum and other psychotic disorders	370 (0.4%)	275 (0.2%)	<.001
Bipolar and related disorders	720 (0.8%)	595 (0.4%)	<.001
Anxiety and fear-related disorders	9825 (10%)	11,015 (7.8%)	<.001
Substance use disorder	12,375 (13%)	7995 (5.6%)	<.001
Charleston comorbidity index	2.00 (2.00, 4.00)	3.00 (2.00, 4.00)	<.001
Dementia	3370 (3.6%)	7695 (5.4%)	<.001
Chronic kidney disease	17,450 (19%)	49,665 (35%)	<.001
Diabetes mellitus	33,285 (35%)	54,075 (38%)	<.001
Essential hypertension	41,755 (44%)	28,755 (20%)	<.001
Hyperlipidemia	65,770 (70%)	104,760 (74%)	<.001
Atrial fibrillation	43,655 (46%)	53,345 (38%)	<.001
Peripheral artery disease	9580 (10%)	23,000 (16%)	<.001
Bicuspid aortic valve	6620 (7.0%)	715 (0.5%)	<.001

Values are n (%) or median (IQR) unless otherwise indicated.

## Discussion

We explored the association between psychiatric conditions and the utilization of TAVR in patients diagnosed with AS. Our findings revealed a significant link between psychiatric comorbidities and a lower likelihood of utilizing TAVR (Central Illustration). This aligns with previous research showing that patients with mental illness are less likely to undergo coronary artery bypass grafting, coronary angioplasty, carotid endarterectomy, and pacemaker placement.^15^, ^16^, ^17^, ^18^, ^19^, ^20^, ^21^ It is crucial to note that patients with various psychiatric conditions, including depressive disorders, anxiety and fear-related disorders, bipolar and related disorders, substance use disorders, and schizophrenia spectrum and other psychotic disorders, all demonstrated a lower likelihood of receiving TAVR compared to patients without these mental health conditions. Furthermore, our study revealed a correlation between the number of psychiatric comorbidities and the probability of undergoing TAVR. Patients with a greater number of psychiatric conditions were associated with a progressively lower likelihood of receiving TAVR. This finding underscores a potential cumulative association of psychiatric comorbidities on utilization of TAVR, suggesting that patients with multiple comorbidities may experience disparities that are even more pronounced.

In our adjusted analysis, factors such as insurance type, female sex, and Black and Asian race were associated with lower odds of TAVR, which is consistent with prior research demonstrating similar disparities.^10^, ^11^, ^12^ These confounders may impact the cohort with psychiatric disorders, as it had a higher number of women and a lower mean age compared to those without psychiatric disorders. Women may have a higher incidence of low-flow and low-gradient AS,^22^ which is more challenging to diagnose and may contribute to the lower utilization of TAVR observed in this subgroup. Moreover, the lower mean age of patients with psychiatric diseases could suggest lower AS severity. Additionally, having AS at a younger age might indicate a higher prevalence of bicuspid aortic valve (BAV). Both the presence of BAV and the younger age itself could influence clinical decisions toward SAVR over TAVR, resulting in lower odds of TAVR.

To assess the impact of race and sex on TAVR utilization, we conducted subpopulation analyses specifically focusing on these 2 factors. This analysis explored the potential interactions between these 2 individual factors and psychiatric comorbidities in TAVR utilization. Our investigation did not reveal any significant interactions between race or sex and psychiatric comorbidities on the likelihood of receiving TAVR. This suggests that the association between psychiatric comorbidities and TAVR utilization is consistent across different racial and sex groups.

To evaluate whether age influenced the results, we adjusted for age in our analysis. This adjustment helps to isolate the impact of psychiatric comorbidities on the likelihood of receiving TAVR, independent of age. Additionally, we utilized the CCI score, which accounts for the age of the patients in evaluating their comorbidity burden, thereby providing a more comprehensive adjustment for the potential influence of age and overall health status on the primary and secondary outcomes of the study.

Furthermore, to determine whether the younger age or a higher prevalence of BAV influenced treatment decisions toward SAVR, we explored the association between psychiatric conditions and the utilization of SAVR. Our findings revealed no significant association between psychiatric disorders and SAVR utilization. We also conducted a post hoc analysis to identify BAV in our data set. The overall prevalence of BAV was 0.7%. Including BAV as a variable in our statistical models did not significantly affect the aOR for TAVR patients with and without psychiatric comorbidities. This suggests that these factors are unlikely to account for the observed differences in TAVR.

The reasons underlying the lower likelihood of TAVR in patients with psychiatric conditions are likely multifaceted. Patient-related barriers may contribute to this disparity. Symptoms of AS, such as fatigue and shortness of breath, might be attributed to or overshadowed by the symptoms of psychiatric conditions.^23^ Additionally, individuals with psychiatric conditions may face unique challenges in compliance, follow-up, access, and engagement with health care. These include limited social support, inadequate understanding of treatment options, concerns related to stigma, and a lack of health care advocacy.^24^, ^25^, ^26^, ^27^ These barriers may lead patients with psychiatric conditions to be more hesitant to undergo invasive procedures such as TAVR or less inclined to actively pursue TAVR as a treatment option. Furthermore, many patients undergoing TAVR are enrolled in clinical trials, and it is possible that psychiatric comorbidities may influence participation in these trials, which could be a contributing factor.

Provider-related factors may also play a role in the observed disparity. Health care providers could harbor implicit biases^28^ or concerns regarding the ability of patients with psychiatric comorbidities to fully comprehend the procedure, provide informed consent, or adhere to postoperative care. Providers might also have reservations about potential complications or increased mortality risk in patients with psychiatric conditions, influencing their decision-making process and willingness to recommend TAVR.^29^, ^30^, ^31^ Additionally, the TAVR procedure mandates a heart team approach, and it is unclear how this multidisciplinary approach impacts treatment decisions for patients with psychiatric comorbidities.

It is crucial to investigate and understand the barriers to guideline-directed treatment modalities for AS in patients with comorbid mental health issues, as the presence of psychiatric disorders could potentially have severe clinical implications. Certain antipsychotic and antidepressant medications are linked to adverse cardiovascular effects, including postural hypotension and arrhythmias, which may exacerbate AS symptoms and pose additional risks.^32^, ^33^, ^34^, ^35^ The anticholinergic effects of specific psychiatric medications, elevating resting heart rates, might further accelerate the progression of aortic valve calcification.^36^ Moreover, therapies like electroconvulsive therapy, frequently used for severe depression, can induce significant hemodynamic changes with potentially devastating consequences in individuals with AS.^37^^,^^38^

Our study bears several notable limitations that warrant consideration in the interpretation of our findings. The retrospective design inherent in our research restricts our ability to definitively establish causality. Using an administrative data set rooted in ICD-10-CM coding and inpatient discharge records introduces inherent constraints. By relying solely on codes from hospital discharges, our study may inadvertently exclude individuals with aortic AS and psychiatric conditions who did not require hospitalization. Furthermore, the inability to distinguish between active and historical psychiatric conditions adds a layer of complexity to our analysis.

One limitation is the inability of the NIS data set to provide information on the severity and clinical characteristics specific to AS. This limits our ability to draw nuanced conclusions regarding treatment patterns and the clinical appropriateness of treatment decisions. Notably, the database may fail to capture subtleties in patient conditions and lacks the granularity necessary for differentiating between comorbidities and complications. This limitation restricts our ability to assess differences in treatment efficacy and TAVR outcomes. Additionally, although we adjusted for important confounders, the under or over-reporting of certain variables such as the presence of BAV in administrative data sets may under or overestimate their impact. Furthermore, the lack of detailed data regarding AS severity, gradient, and flow rate might introduce confounders unaccounted for in our analysis, thereby affecting the observed differences.

Moreover, the limitations in patient identification and data capture across various health care settings within the NIS database may introduce bias into our analysis. Treating multiple hospitalizations for the same patient as distinct observations could lead to potential overestimation or underestimation of certain measures and outcomes. The NIS might also overlook health care encounters or procedures occurring outside the hospital setting, potentially introducing bias by omitting crucial information.

Despite these limitations, it is helpful to acknowledge the strengths of our study. Our research benefits from a large sample size with minimal excluded data, and previous studies have validated the reliability of ICD code-based data sets, like ours, for psychiatric diagnoses, demonstrating high specificity and moderate sensitivity compared to chart reviews.^39^ Additionally, we implemented statistical methods to account for unmeasured confounders and excluded data. Although interpreting retrospective designs demands caution, the strengths may allow for inferences from the data. Future research with longitudinal designs and registry-based studies that include detailed information on AS characteristics and echocardiographic parameters could help clarify the associations observed and potentially establish a causal relationship. Qualitative research could identify reasons for the underutilization of TAVR in those with psychiatric disorders and offer areas for intervention. Additionally, interventional studies could investigate how effective management of psychiatric disorders with current guideline therapy might influence TAVR utilization and the outcome.

## Conclusion

Our study provides compelling observational evidence of a significant potential association between psychiatric conditions and reduced utilization of TAVR in patients diagnosed with AS. These findings underscore the imperative for further research aimed at confirming our observations and comprehensively exploring the factors contributing to these disparities and devising strategies to effectively address them.

## Declaration of artificial intelligence (AI) and AI-assisted technologies in the writing process

During the preparation of this work, the authors used Grammerly AI and OpenAI in order to correct grammar and spelling. After using this tool/service, the authors reviewed and edited the content as needed and take full responsibility for the content of the publication.
