# Supplementary material for: Differences in TAVR Utilization in Aortic Stenosis Among Patients With and Without Psychiatric Comorbidities
Source: J Soc Cardiovasc Angiogr Interv. 2024 Aug 13;3(9):102235. doi: 10.1016/j.jscai.2024.102235 (PMC11576367; doi:10.1016/j.jscai.2024.102235)
Supplement: Supplemental Material [file mmc1.docx]

**Supplementary Appendix**

**Treatment Disparities and TAVR Utilization in Aortic Stenosis Among Patients with Psychiatric Comorbidities**

| Description | Content |
| --- | --- |
| ICD-10-CM codes for cohort identification and comorbidities | Table S1 |
| Matching variables used in multivariate logistics regression. | Table S2 |
| Distribution of ICD-10 Codes for Aortic Stenosis | Table S3 |
| Forest plot showing the adjusted Odds Ratios of TAVR based on the burden of psychiatric comorbidities | Figure S1 |
| Methodology for Psychiatric Disorders Analysis | Section 1 |
| Sample of logistic regression models used | Section 2 |

**Abbreviations used in the manuscript and supplementary file:**

**aOR =** Adjusted Odds Ratio; **AS =** Aortic Stenosis; **CABG =** Coronary Artery Bypass Graft; **CCS =** Clinical Classification Software; **ICD-10-CM =** International Classification of Diseases, Tenth Revision, Clinical Modification; **NIS =** National Inpatient Sample; **PCI =** Percutaneous Coronary; **SAVR =** Surgical Aortic Valve Replacement, Intervention; **TAVR =** Transcatheter Aortic Valve Replacement; **TPA =** Tissue Plasminogen Activator.

**Table S1:** ICD-10-CM codes of major variables in the study

| Variable | ICD-10-CM diagnosis codes |
| --- | --- |
| Alcohol Use Disorders | F1010, F10120, F10121, F10129, F1014, F10150, F10151, F10159, F10180, F10181, F10182, F10188, F1019, F1020, F10220, F10221, F10229, F10230, F10231, F10232, F10239, F1024, F10250, F10251, F10259, F1026, F1027, F10280, F10281, F10282, F10288, F1029, F10920, F10921, F10929, F1094, F10950, F10951, F10959, F1096, F1097, F10980, F10981, F10982, F10988, F1099, G312, |
| Nicotine and Tobacco Use disorder | Z720, F17200, F17203, F17208, F17209, F17210, F17213, F17218, F17219, F17220, F17223, F17228, F17229, F17290, F17293, F17298, F17299 |
| Substance Use disorder | F1210, F12120, F12121, F12122, F12129, F12150, F12151, F12159, F12180, F12188, F1219, F1220, F12220, F12221, F12222, F12229, F1223, F12250, F12251, F12259, F12280, F12288, F1229, F1290, F12920, F12921, F12922, F12929, F1293, F12950, F12951, F12959, F12980, F12988, F1299, T407X1A, T407X1D, T407X2A, T407X2D, T407X3A, T407X3D, T407X4A, T407X4D, T407X5A, T407X5D, , F1110, F11120, F11121, F11122, F11129, F1114, F11150, F11151, F11159, F11181, F11182, F11188, F1119, F1120, F11220, F11221, F11222, F11229, F1123, F1124, F11250, F11251, F11259, F11281, F11282, F11288, F1129, F1190, F11920, F11921, F11922, F11929, F1193, F1194, F11950, F11951, F11959, F11981, F11982, F11988, F1199, T400X1A, T400X1D, T400X2A, T400X2D, T400X3A, T400X3D, T400X4A, T400X4D, T400X5A, T400X5D, T401X1A, T401X1D, T401X2A, T401X2D, T401X3A, T401X3D, T401X4A, T401X4D, T402X1A, T402X1D, T402X2A, T402X2D, T402X3A, T402X3D, T402X4A, T402X4D, T402X5A, T402X5D, T403X1A, T403X1D, T403X2A, T403X2D, T403X3A, T403X3D, T403X4A, T403X4D, T403X5A, T403X5D, T404X1A, T404X1D, T404X2A, T404X2D, T404X3A, T404X3D, T404X4A, T404X4D, T404X5A, T404X5D, T40601A, T40601D, T40602A, T40602D, T40603A, T40603D, T40604A, T40604D, T40605A, T40605D, T40691A, T40691D, T40692A, T40692D, T40693A, T40693D, T40694A, T40694D, T40695A, T40695D, F1410, F14120, F14121, F14122, F14129, F1414, F14150, F14151, F14159, F14180, F14181, F14182, F14188, F1419, F1420, F14220, F14221, F14222, F14229, F1423, F1424, F14250, F14251, F14259, F14280, F14281, F14282, F14288, F1429, F1490, F14920, F14921, F14922, F14929, F1494, F14950, F14951, F14959, F14980, F14981, F14982, F14988, F1499, F1510, F15120, F15121, F15122, F15129, F1514, F15150, F15151, F15159, F15180, F15181, F15182, F15188, F1519, F1520, F15220, F15221, F15222, F15229, F1523, F1524, F15250, F15251, F15259, F15280, F15281, F15282, F15288, F1529, F1590, F15920, F15921, F15922, F15929, F1593, F1594, F15950, F15951, F15959, F15980, F15981, F15982, F15988, F1599, T405X1A, T405X1D, T405X2A, T405X2D, T405X3A, T405X3D, T405X4A, T405X4D, T405X5A, T405X5D, T43601A, T43601D, T43602A, T43602D, T43603A, T43603D, T43604A, T43604D, T43605A, T43605D, T43611A, T43611D, T43612A, T43612D, T43613A, T43613D, T43614A, T43614D, T43615A, T43615D, T43621A, T43621D, T43622A, T43622D, T43623A, T43623D, T43624A, T43624D, T43625A, T43625D, T43631A, T43631D, T43632A, T43632D, T43633A, T43633D, T43634A, T43634D, T43635A, T43635D, T43641A, T43641D, T43642A, T43642D, T43643A, T43643D, T43644A, T43644D, T43691A, T43691D, T43692A, T43692D, T43693A, T43693D, T43694A, T43694D, T43695A, T43695D |
| Depressive Disorders | F0631, F0632, F0634, F320, F321, F322, F323, F324, F328, F3281, F3289, F329, F330, F331, F332, F333, F3341, F338, F339, F341 |
| Bipolar and Related Disorders | F0633, F0634, F3010, F3011, F3012, F3013, F302, F303, F308, F309, F310, F3110, F3111, F3112, F3113, F312, F3130, F3131, F3132, F314, F315, F3160, F3161, F3162, F3163, F3164, F3171, F3173, F3175, F3177, F3181, F3189, F319, F340 |
| Anxiety and Fear Related Disorders | F064, F10180, F10280, F10980, F12180, F12280, F12980, F13180, F13280, F13980, F14180, F14280, F14980, F15180, F15280, F15980, F16180, F16280, F16980, F18180, F18280, F18980, F19180, F19280, F19980, F4000, F4001, F4002, F4010, F4011, F40210, F40218, F40220, F40228, F40230, F40231, F40232, F40233, F40240, F40241, F40242, F40243, F40248, F40290, F40291, F40298, F408, F409, F410, F411, F413, F418, F419, F930, F940 |
| Schizophrenia Spectrum and Other Psychotic Disorders | F060, F061, F062, F10150, F10151, F10159, F10250, F10251, F10259, F10950, F10951, F10959, F11150, F11151, F11159, F11250, F11251, F11259, F11950, F11951, F11959, F12150, F12151, F12159, F12250, F12251, F12259, F12950, F12951, F12959, F13150, F13151, F13159, F13250, F13251, F13259, F13950, F13951, F13959, F14150, F14151, F14159, F14250, F14251, F14259, F14950, F14951, F14959, F15150, F15151, F15159, F15250, F15251, F15259, F15950, F15951, F15959, F16150, F16151, F16159, F16250, F16251, F16259, F16950, F16951, F16959, F18150, F18151, F18159, F18250, F18251, F18259, F18950, F18951, F18959, F19150, F19151, F19159, F19250, F19251, F19259, F19950, F19951, F19959, F200, F201, F202, F203, F205, F2081, F2089, F209, F21, F22, F23, F24, F250, F251, F258, F259, F28, F29 |
| Dementia | D631, E0822, E0922, E1022, E1122, E1322, I120, I129, I130, I1310, I1311, I132, N181, N182, N183, N184, N185, N186, N189, R880, Z4901, Z4902, Z4931, Z4932 |
| Aortic Stenosis | I350, I352, I358 |
| Chronic Kidney Disease | D631, E0822, E0922, E1022, E1122, E1322, I120, I129, I130, I1310, I1311, I132, N181, N182, N183, N184, N185, N186, N189, R880, Z4901, Z4902, Z4931, Z4932 |
| Diabetes Mellitus | E08, E11, E13 |
| Essential Hypertension | I10 |
| Hyperlipidemia | E78 |
| Atrial Fibrillation | I48 |
| Peripheral Artery Disease | I739, I70 |
| TAVR ICD-10-PCS codes | 02RF38H, 02RF38Z, 02RF3KH, 02RF3KZ |
| SAVR ICD-10-PCS codes | 02RF07Z, 02RF08Z, 02RF0JZ, 02RF0KZ |
| Bicuspid Aortic Valve | Q231 |

**Table S2:** Matching variables used in multivariate logistics regression

| Age |
| --- |
| Gender |
| Race |
| Payer Type |
| Type of Admission |
| Hospital Region |
| Hospital Control |
| Hospital Teaching Status and Location |
| Hospital Bed Size |
| Household Income Quartile |
| Charleston Comorbidity Index |
| Dementia |
| Chronic Kidney disease |
| Diabetes Mellitus |
| Essential Hypertension |
| Hyperlipidemia |
| Atrial Fibrillation |
| Peripheral Artery Disease |

**Table S3:** Distribution of ICD-10 Codes for Aortic Stenosis

| AS ICD-10 Codes | Overall, n(%)* | TAVR, n(%)^+^ | SAVR, n(%)^+^ |
| --- | --- | --- | --- |
| I350 | 1,385,620 (89%) | 129,095 (9.3%) | 77,595 (5.6%) |
| I352 | 85,565 (5.5%) | 12,900 (15.1%) | 15,340 (17.9%) |
| I358 | 78,600 (5.1%) | 105 (0.1%) | 1,310 (1.7%) |

Abbreviation: AS = Aortic Stenosis; SAVR = Surgical Aortic Valve Replacement; TAVR = Transcatheter Aortic Valve Replacement. * Represents the total number and percentage of each AS ICD-10 code in the sample. ^+^ Represents the number and percentage of TAVRs and SAVRs in each ICD-10 code

**Figure S1:** Forest plot showing the adjusted Odds Ratios of TAVR based on the burden of psychiatric comorbidities


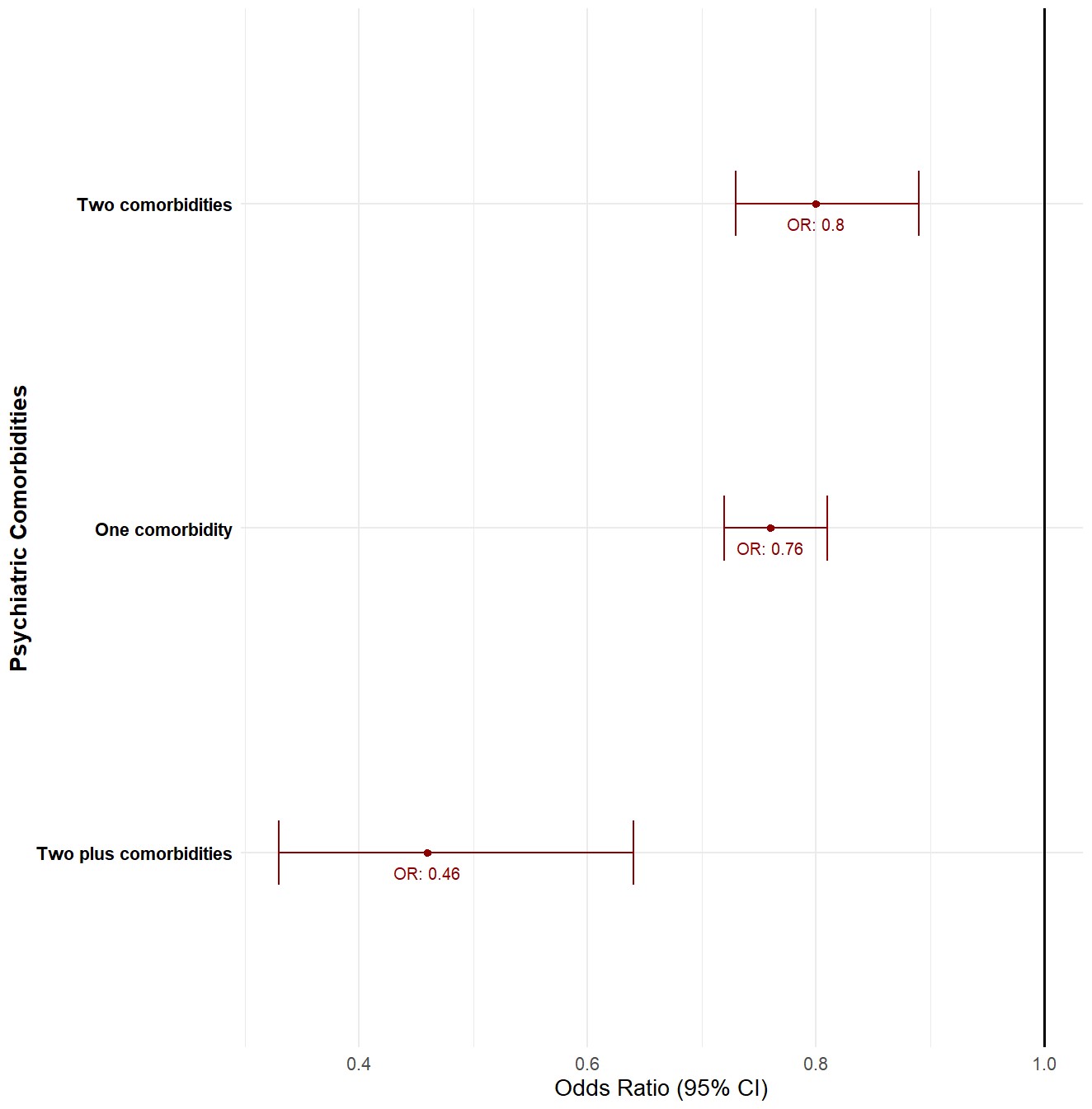


Adjusted multivariable logistic regression models for TAVR in AS patients based on the burden of psychiatric comorbidities, relative to those without any psychiatric conditions.

CI = confidence interval, OR = Odds Ratio

**Section 1: Methodology for Psychiatric Disorders Analysis**

1. **Define Subset for Psychiatric Disorders Analysis:**

- Set AS_1 as a subset of the main dataset AS. This subset should include only columns related to psychiatric disorders, ranging from ‘Depressive_disorders’ to ‘Substance_use_disorder’.
- Note: Each disorder is represented by a set of specific diagnostic codes. The presence of any of these codes in a patient’s data indicates a diagnosis for that particular disorder.

1. **Calculate Psychiatric Comorbidity Indicators:**

- Initialize an empty structure, psych_comorbidities.
- For each patient (row) in AS_1, perform the following:
  1. Count the total number of diagnosed psychiatric disorders and store this count in a temporary variable x.
  2. Determine if the patient has at least one psychiatric disorder (at_least_one_psych), setting it to TRUE if x > 0, else FALSE.
  3. Determine if the patient has exactly one disorder (one_psych), setting it to TRUE if x == 1, else FALSE.
  4. Determine if the patient has exactly two disorders (two_psych), setting it to TRUE if x == 2, else FALSE.
  5. Determine if the patient has more than two disorders (two_plus_psych), setting it to TRUE if x > 2, else FALSE.

1. **Convert to Tabular Format and Merge with Original Dataset:**

- Convert the psych_comorbidities structure into a tabular format akin to a data frame.
- Select only the newly created indicators for psychiatric disorders within this structure.
- Merge these indicators as new columns into the original dataset AS, thus including indicators for the presence and count of psychiatric disorders for each patient.

1. **Categorize Patients Based on Number of Psychiatric Comorbidities:**

- For each patient in AS, create a new variable Number_of_psychiatric_comorbidities.
- Categorize each patient based on the number of psychiatric disorders:
  1. Label as “no psychiatric comorbidities” if at_least_one_psych is FALSE.
  2. Label as “one comorbidity” if one_psych is TRUE.
  3. Label as “two comorbidities” if two_psych is TRUE.
  4. Label as “two plus comorbidities” only if two_plus_psych is TRUE and two_psych is FALSE.

**Note on Patient Diagnosis with Multiple Diagnostic Code Matches:**

- In the context of a Clinical Classifications Software System, it’s crucial to understand the interpretation of multiple diagnostic codes.
- A patient may have several codes within the same clinical classification category (e.g., different types of depressive disorders).
- For the purpose of categorization:
  1. A patient with multiple diagnostic codes within the same category is labeled as having a single psychiatric comorbidity for that group.
  2. A patient is classified as having “two comorbidities” only if they have diagnostic codes that match two separate diagnostic categories.
  3. A patient is classified as having “two plus comorbidities” if they have codes from three or more different clinical classification groups.
- This approach ensures that multiple codes within the same category are consolidated into a single indicator of presence for that psychiatric condition.

**Section 2: Statistical analysis and Sample of logistic regression models used**

**Statistical Analysis**

All statistical analyses in this study were performed using the R software (version 4.2.3, R Foundation for Statistical Computing, Vienna, Austria). To specifically handle and analyze the complex survey data, we employed the 'survey' package (Lumley, 2023). This package is designed for analysis of complex survey samples and is well-suited for the methodologies employed in our study.

**References**

- - 1. <https://hcup-us.ahrq.gov/db/nation/nis/nischecklist.jsp>
    2. <https://hcup-us.ahrq.gov/db/nation/nis/nisdbdocumentation.jsp>
    3. Lumley T (2023). "survey: analysis of complex survey samples." R package version 4.2.
    4. Lumley T (2004). “Analysis of Complex Survey Samples.” Journal of Statistical Software, 9(1), 1-19.
    5. Lumley T (2010). Complex Surveys: A Guide to Analysis Using R. John Wiley and Sons.

**Model 1: Binary Psychiatric Comorbidity**

- Model Name: logit1
- Purpose: Assessing the impact of any psychiatric comorbidity on TAVR likelihood.
- Key Predictor: Binary coded Number_of_psychiatric_collapsed.
- Interactions: Sex*Number_of_psychiatric_collapsed, Race*Number_of_psychiatric_collapsed.
- Other Predictors:
  - Demographic: Age, Sex, Race
  - Health Comorbidities: Charleston_comorbidity_index, Dementia, Chronic_kidney_disease, Diabetes_mellitus, Essential_hypertension, Hyperlipidemia, Atrial_fibrillation, Peripheral_Artery_Disease
  - Hospital Factors: Insurance, Type_of_admission, Hospital_region, Hospital_control, Hospital_teaching_status_and_location, Hospital_bed_size
  - Socioeconomic: Household_income_quartile

**Model 2: Mutually Exclusive Psychiatric Comorbidities**

- Model Name: logit2
- Purpose: Evaluating the effect of psychiatric comorbidities, treated as mutually exclusive, on TAVR.
- Key Predictor: Psychiatric_comorbidities_mutually_exclusive.
- Other Predictors:
  - Demographic: Age, Sex, Race
  - Hospital Factors: Insurance, Type_of_admission, Hospital_region, Hospital_control, Hospital_teaching_status_and_location, Hospital_bed_size
  - Health Comorbidities: Charleston_comorbidity_index, Dementia, Chronic_kidney_disease, Diabetes_mellitus, Essential_hypertension, Hyperlipidemia, Atrial_fibrillation, Peripheral_Artery_Disease
  - Socioeconomic: Household_income_quartile

**Model 3: Interaction with Number of Psychiatric Comorbidities**

- Model Name: logit3
- Purpose: Analyzing the interaction effects of Sex and Race with the number of psychiatric comorbidities on TAVR.
- Key Predictor: Number_of_psychiatric_comorbidities.
- Interactions: Sex*Number_of_psychiatric_comorbidities, Race*Number_of_psychiatric_comorbidities.
- Other Predictors:
  - Demographic: Age, Sex, Race
  - Hospital Factors: Insurance, Type_of_admission, Hospital_region, Hospital_control, Hospital_teaching_status_and_location, Hospital_bed_size
  - Health Comorbidities: Charleston_comorbidity_index, Dementia, Chronic_kidney_disease, Diabetes_mellitus, Essential_hypertension, Hyperlipidemia, Atrial_fibrillation, Peripheral_Artery_Disease
  - Socioeconomic: Household_income_quartile

**Model 4: Individual Psychiatric Comorbidities**

- Model Name: logit4
- Purpose: Investigating the impact of specific psychiatric disorders on TAVR.
- Key Predictors: Depressive_disorders, Schizophrenia_spectrum_and_other_psychotic_disorders, Bipolar_and_related_disorders, Anxiety_and_fear_related_disorders, Substance_use_disorder.
- Other Predictors:
  - Demographic: Age, Sex, Race
  - Hospital Factors: Insurance, Type_of_admission, Hospital_region, Hospital_control, Hospital_teaching_status_and_location, Hospital_bed_size
  - Health Comorbidities: Charleston_comorbidity_index, Dementia, Chronic_kidney_disease, Diabetes_mellitus, Essential_hypertension, Hyperlipidemia, Atrial_fibrillation, Peripheral_Artery_Disease
  - Socioeconomic: Household_income_quartile

Each model uses a quasibinomial family and is adjusted using a survey design specified by AS.survey. These models are distinct in their approach to psychiatric comorbidities, allowing for a comprehensive analysis of their impact on TAVR.
